# Effect of Salt Mist Ageing on the Physical and Mechanical Properties of Two Ignimbrites from the Canary Islands (Spain)

**DOI:** 10.3390/ma16227061

**Published:** 2023-11-07

**Authors:** José A. Valido, José M. Cáceres, D. M. Freire-Lista, Luís M. O. Sousa

**Affiliations:** 1 Department of Industrial Engineering, University of La Laguna (ULL), Campus Ancheta, 38200 La Laguna, Spain; jvalidog@ull.edu.es (J.A.V.); jmcacer@ull.edu.es (J.M.C.); 2 Department of Geology, University of Trás-os-Montes e Alto Douro (UTAD), Quinta de Prados, 5001-801 Vila Real, Portugal; davidfreire@utad.pt; 3 CGeo–Geosciences Center, University of Coimbra, Rua Silvino Lima, Polo II, 3030-790 Coimbra, Portugal

**Keywords:** natural stone, building stone, ignimbrite, salt mist, weathering, ageing tests

## Abstract

The aim of this research work is to study the effect of salt mist ageing on the physico-mechanical properties of two ignimbrites from the Canary Islands (Spain). Due to their insular nature and extensive coastlines, these regions are highly susceptible to atmospheric salt aerosols, which is a significant weathering agent for building materials. The ignimbrites analysed are marketed under the names “Azul Lomo Tomás de León” and “Marrón de Abades” and are widely used as building stones. Petrographic, mineralogical and chemical properties were obtained via polarisation microscopy, X-ray diffraction and X-ray fluorescence. The samples were subjected to 60 cycles of a salt mist environment, following relevant European standards. Scanning electron microscopy images of the specimens were analysed along with physical properties, including apparent density, open porosity, water absorption, colourimetry and ultrasound propagation velocity, initially and after every 20 cycles, allowing us to assess their evolution during the ageing test. Mechanical properties were evaluated using uniaxial compressive strength and flexural strength under concentrated load tests on two groups of specimens: one unaged and the other subjected to 60 ageing cycles. With the exception of apparent density, the other properties show a decrease between 5% and 30%. However, open porosity increases and is one of the properties most affected by the ageing test.

## 1. Introduction

Natural stone, when used as a building material, must resist deterioration, fulfilling its functions after long periods of use. Deterioration, also known as decay, weathering or degradation, is a process that refers to the altered state of stones [1]. It can be divided into three types, chemical, physical and biological (biodeterioration) [2], depending on whether it is mainly caused by air pollution, frost, soluble salts, temperature changes, alternate wetting and drying or the action of biological agents [3]. The ability of stones to resist deterioration while retaining their original appearance and strength is known as durability [4]. It is very important for the preservation and conservation of the architectural heritage [5]. It also plays a fundamental role in modern architecture, as natural stones are still widely used in today’s buildings [6].

Durability can be determined indirectly through the analysis of petrographic and physicomechanical properties [7,8] or directly through ageing tests.

### 1.1. Indirect Determination of Durability

(i) Petrographic properties: The mineral’s size and shape determine the deterioration behaviour of stones. Clay minerals associated with sedimentary rocks or the secondary minerals, either in endogenous processes (hydrothermal or deuteric alteration) or in exogenous processes (meteoric alteration), have a negative influence on durability [9]. The presence of anisotropy can also determine the degree of durability.
(ii) Physical properties: These are often used to characterise durability, especially those related to water behaviour, as water is one of the main elements in the deterioration of building stones [10]. Porosity, together with pore size and pore distribution, is one of the most influential properties on durability [11,12]. A good indicator of the internal effects of deterioration is also the ultrasound propagation velocity [13].
(iii) Mechanical properties: These are one of the most important properties to characterise durability, as the mechanical strength of stones is related to the degree of cohesion [14]. Although different mechanisms can lead to stone deterioration, the most frequent cause is periods of expansion and contraction that create compressive and tensile loads [15]. These loads are particularly critical when exceeding the cohesive forces. Among the available mechanical tests, compressive strength test seems to be the best parameter to determine the degree of deterioration [16]. In general, stones with a higher mechanical strength have higher durability and vice versa [17].

### 1.2. Direct Determination of Durability by Ageing Tests

During these tests, the samples are subjected to an accelerated artificial ageing process in which they are exposed to pollutants like sulphates and nitrates, salts or ultraviolet radiation, or may be subjected to rapid heating–cooling, freeze–thawing or wetting–drying cycles [18]. It is important to note that ageing test conditions are typically more severe than natural exposures [19]. The purpose of these tests is to determine how the building stone will perform in a specific environmental condition or to evaluate the effectiveness of a protective treatment in preventing deterioration. Frost resistance, salt crystallisation resistance and thermal shock ageing resistance are the most frequent tests used to assess the durability of natural stones [20]. However, other tests such as resistance to SO_2_ ageing, wetting–drying cycles and salt mist ageing resistance are widely used. Salt mist ageing tests are particularly useful in reproducing the natural weathering conditions found in coastal areas [21].

### 1.3. Background

Salts are a major cause of deterioration of building stones [22], which can occur by different mechanisms depending on the crystallisation type [23]. Experimentally, to examine the impact of these weathering processes associated with soluble salts on petrographic, physical and mechanical properties, various ageing tests can be conducted experimentally to simulate the deterioration. Salt crystallisation resistance [24] and salt mist resistance [25] are the most commonly used tests. Although relevant standards provide test specifications and requirements, researchers have a tendency to vary the test conditions, such as sample size, type and concentration of saline solution, temperature, number of cycles, etc. [26,27].

Based on the consulted literature, limestone is one of the most studied building stones in terms of deterioration caused by salt mist. Pires et al. [28,29] studied two limestones from Portugal and concluded that after exposure to 45 cycles of salt spray, the samples suffered a loss of mass (between 4.0 and 9.0%) as well as a reduction in modulus of elasticity (between 20 and 30%) and in flexural strength (close to 30%). The behaviour of a group of six limestones, also from Portugal, was studied Carvalho et al. [30,31]. After 60 cycles of salt spray ageing, the loss of mass is not very significant (between 0.1 and 0.4%) and the water absorption is hardly changed. The results on flexural strength are not very conclusive, because while some samples suffer deterioration (between 4.0 and 12.0%), others increase their strength (between 4.0 and 8.0%). In their study of three Portuguese limestones, Simão et al. [32] obtained a practically negligible mass loss (between 0.0 and 0.07%) after 60 salt mist cycles. Fort et al. [33] subjected a limestone quarried in Spain to 120 salt mist cycles, and found that apparent density increased by 0.3%, open porosity and water absorption were unchanged and ultrasound propagation velocity decreased by 4.4%. According to their results, colour and roughness were the properties that showed the greatest variation. Karakaş et al. [34] obtained a mass loss of 0.08% in a limestone from Turkey, despite the fact that they used a calcium chloride (CaCl_2_) solution instead of the sodium chloride (NaCl) solution as recommended in the standard.

Kłopotowska and Łukaszewski [35] investigated the influence of 60 salt mist cycles on two limestones and two sandstones, all from Poland. Limestones had a mass loss of less than 1.0%, while sandstones varied between 0.5 and 2.0%. The ultrasound velocity decreased in all samples (between 3.0 and 9.0%). Compressive strength, deformation and elastic modules were also evaluated in this study. Some samples showed a decrease in compressive strength up to 30%. The deformation for the sandstones increased up to 40%, while for the limestones, it decreased. With regard to the modulus of elasticity, it decreased between 7 and 21% in all samples except in one of the limestones. In a group of four sandstones from Morocco, Ouacha et al. [36] found mass losses between 0.1 and 0.9%, except in one whose mass increased by 0.2%. Çelik et al. obtained a mass loss of 0.49% after 60 salt mist cycles [37] in their study of an andesite from Turkey. Valido et al. [38] obtained a mass loss varying between 0.04 and 0.10% in two ignimbrites from the Canary Islands (Spain).

Borges et al. [39] artificially aged five granites quarried in different regions of Portugal and, after being exposed to 150 cycles of salt mist, concluded that deterioration caused a decrease in apparent density (between 0.1 and 0.9%) and compressive strength (between 7.0 and 62%), while open porosity and water absorption increased. The mean mass loss over 60 cycles was approximately 0.15%. Oliveira [40] studied the weathering of a trachyte extracted in Portugal by carrying out various ageing tests. To evaluate the effects of salt mist, the specimens were subjected to 226 cycles, and the researchers found that open porosity, water absorption and compressive strength decreased by 48%, 30% and 25%, respectively, while ultrasonic velocity increased by 13%. Mass loss was less than 1.0%.

Silva and Simão [41] subjected a group of silicate rocks (granites, syenites, gabbro and basalt) and carbonate rocks (limestones and marbles), commonly used as building stones in Portugal and Brazil, to 14 modified ageing cycles. One cycle corresponded to ten days of exposure (five sub-cycles of 12 h of salt mist and 36 h of drying) and the salt solution had a concentration of 180 g/L (the standard proposes a concentration of 100 g/L). The mass loss of silicate rocks varied between 0.05 and 0.70%, while carbonate rocks was much higher, between 0.05 and 5.5%. Silva et al. [42] applied the same procedure to a group of silicate rocks (granite, anorthosite and nepheline syenite) with different surface finishes (polished, honed, hammered and flamed). They concluded that polished samples had the lowest mass loss, while hammered samples had the highest. Leal et al. [43] also divided their study samples into silicate rocks (one nepheline syenite and one granite) and carbonate rocks (three limestones) in their research to evaluate the effectiveness of two conservation treatments (one hydrophobic and one consolidating). They concluded that the treatments protected the stones, although their effectiveness was relative because the treatments changed the colour of the samples after six salt mist cycles. Urosevic et al. [44] focused on analysing the deterioration caused by salt spray as a function of surface finish (polished and rough) in a travertine quarried in southern Spain. They also concluded that deterioration was lower on the polished surface after subjecting the samples to 160 cycles (3 h of salt spray and 6 h of drying).

Costa and Rodrigues [45] studied, in addition to mass loss, the effect of salt weathering on drilling resistance by subjecting different types of stones (marble, limestone and sandstone) to 50 salt mist cycles (10 h salt spray and 38 h drying). The sandstone had the highest mass loss variation (between 1.69 and 2.22%) and marble had the lowest (between 0.07 and 0.11%). As far as drilling resistance is concerned, resistance decreased near the surface, but differences were barely noticeable inside the samples.

In the research mentioned above, porosity stands out as a key factor in the durability of the stones. Stones with a higher porosity are more likely to retain salt crystals and are therefore more affected by the salt mist ageing test. The present work studies the effect of salt mist on the physical–mechanical properties of two ignimbrites from the Canary Islands (Spain), as should be performed in any systematic inventory and evaluation of stone resources [46]. The analyses include petrographic, mineralogical and chemical characterisation. The interest in using the accelerated salt mist ageing test is to simulate the deterioration that could occur when stones are exposed to marine aerosols, which is significant in island regions. The aim of this work is to assess and understand the salt weathering of ignimbrites, for which little information is available. For this purpose, the apparent density, open porosity, water absorption, colorimetric parameters, ultrasonic velocity, compressive strength and flexural strength were determined initially and after 60 salt spray cycles. In addition, the variation of the physical properties as a function of the number of cycles has been studied and determined every 20 cycles.

## 2. Materials

**Figure 1:**
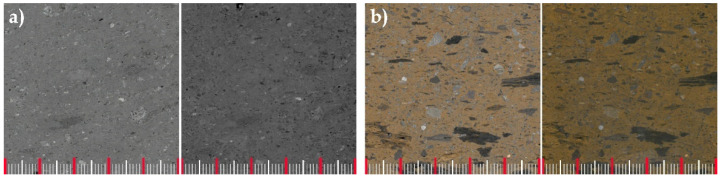
Macroscopic appearance of sawn surfaces ignimbrites: (**a**) Lomo Tomás de León (LT) and (**b**) Marrón de Abades (MR). Dry surface (**left**); wet surface (**right**). Distance between red lines: 10 mm.

Two ignimbrites from the Canary Islands (Spain) have been selected: Lomo Tomás de León (LT) and Marrón de Abades (MR). The LT ignimbrite is mined in the north of Gran Canaria and belongs to the “Arucas ignimbritic breccia” geological unit. This geological unit is made up of part of the material found in the explosive eruptions that took place between 13.3 and 8.3 Ma (Miocene), whose differentiated felsic magmas were characterised by a trachyte–phonolitic composition [47]. This period corresponds to the final stages of the construction episode, Cycle I [48]. The LT ignimbrite is bluish-grey in colour, its fragments are not very prominent, and the direction of flow is not highly marked (Figure 1a). MR ignimbrite is mined in the south–east of Tenerife and belongs to the “Ignimbritas de Arico” geological unit. This geological unit, which also includes “Piedra Chasnera” [49], is made up of highly viscous phonolitic flows with a high volatile content, resulting from the gravitational or explosive collapse of a dome [50]. The geological age of this unit is estimated to be 0.65 Ma (Middle Pleistocene) [51], corresponding to the constructive episode of the Tenerife island, Cañadas III [52]. The MR ignimbrite is ochre coloured with visible lithic fragments, mostly elongated in the direction of flow (Figure 1b).

## 3. Methods

### 3.1. Petrographic, Mineralogical and Chemical Analysis

Petrographic properties were determined according to the specifications of EN 12407 [53]. A thin section (30 µm × 45 mm × 25 mm) per sample was prepared and examined with a Leica DM750P optical microscope. The chemical composition was determined via X-ray fluorescence (XRF) analysis according to EN 15309 [54] and was performed by the laboratory personnel of Activation Laboratories Ltd. (Hamilton, ON, Canada) through wavelength dispersive X-ray fluorescence (WDXRF). Major element (oxide) analysis was carried out using the heavy absorber fusion technique of Norrish and Hutton [55] and the loss on ignition (LOI) was determined from the weight loss after roasting the sample at 1000 °C for 2 h. Mineralogical analysis via X-ray diffraction (XRD) was carried out at the Electronic Microscopy Unit (UME) of Trás-os-Montes e Alto Douro University (UTAD) and was performed at room temperature using a PANalytical X’Pert Pro diffractometer equipped with an X’Celerator detector and a secondary monochromator. Samples were prepared for a standard powder sample holder using the inverted sample method. The energy used to generate the X-rays was 40 kV and 30 mA. The acquisition was performed in Bragg-Brentano geometry between 7° < 2θ < 80°, with CuKα radiation (λα_1_ = 1.54060 Å and λα_2_ = 1.54443 Å, 0.017°/step, 100 s/step). The phases obtained were identified using the software HighScore Plus 4.8. and PowderCell 2.4. with International Centre for Diffraction Data (ICDD) and Crystallography Open Database (COD) databases. Semi-quantifications were conducted using Rietveld refinements. Scanning electron microscopy (SEM) was also performed at the UME, using an SEM FEI Quanta 400 scanning electron microscope with tungsten (W) hairpin filament and with environmental SEM/E-SEM and chemical analysis via EDS (EDAX), with a resolution of 4 nm and operated with xT Microscope Control Software, version 2.4, FEI Company (Hillsboro, OR, USA).

### 3.2. Physical Properties

On the basis of the test methods described in the relevant European standards, the following physical properties were determined: apparent density and open porosity (EN 1936) [56], water absorption at atmospheric pressure (EN 13755) [57], P-wave ultrasound propagation velocity (EN 14579) [58] and colour (EN 15886) [59]. All these physical properties were determined on the same group of six cubic specimens of dimensions (50 × 50 × 50) mm. The ultrasound propagation velocity corresponds to the mean value measured in the three directions (X, Y, Z). Pundit Lab+ equipped with 54 kHz transducers from Screening Eagle was used. The colour corresponds to the mean value of the parameters obtained in each test specimen measured on one side of the cube following a (3 × 3) matrix. Colour is expressed using the CIEL*a*b* systems and measured with an X-Rite spectrophotometer (model 964). Ultrasound propagation velocity and colour were measured in both dry and wet conditions.

### 3.3. Mechanical Properties

**Figure 2:**
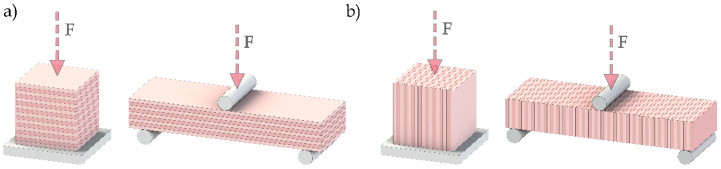
Schematic representation of oriented sample tests used for the mechanical tests: (**a**) load perpendicular to the anisotropy planes (Z) and (**b**) load parallel to the anisotropy planes (Y).

The mechanical properties determined were: uniaxial compressive strength (EN 1926) [60] and flexural strength under concentrated load (EN 12372) [61]. Twenty (20) specimens were used in each of these tests. For uniaxial compressive strength, the specimens were cubes (50 × 50 × 50 mm), while for flexural strength, specimens with dimensions 180 × 60 × 30 mm were used. For both uniaxial compressive strength and flexural strength tests, the specimens were divided into two groups (10 specimens in each group). In one group, the load was applied perpendicular to the anisotropy planes (Z) (Figure 2a) and in the other, the load was applied parallel to the anisotropy planes (Y) (Figure 2b). The uniaxial compressive strength and flexural strength results correspond to the mean value of both directions. The compression tests have been carried out using a test frame equipped with an actuator with a load capacity of 3000 kN and the flexural tests were performed on an MTS universal testing machine, model Exceed E45 with 100 kN load capacity.

### 3.4. Ageing by Salt Mist

Salt mist ageing process was carried out according to the methodology described in the European standard EN 14147 [25]; although this standard was withdrawn in 2019, it is still one of the most widely used methods in the literature to simulate exposure to saline atmospheres. According to this standard, the specimens were subjected to a total of 60 cycles (4 h ± 15 min of salt mist—sodium chloride solution with a concentration of 100 ± 10 g/L– and 8 h ± 15 min of drying) in a salt mist chamber (Corrosionbox, model CRBX 400, from Cofomegra was used) at a temperature of 35 ± 5 °C, but the number of specimens used and their dimensions were modified according to the physical and mechanical properties to be determined after ageing.

**Figure 3:**
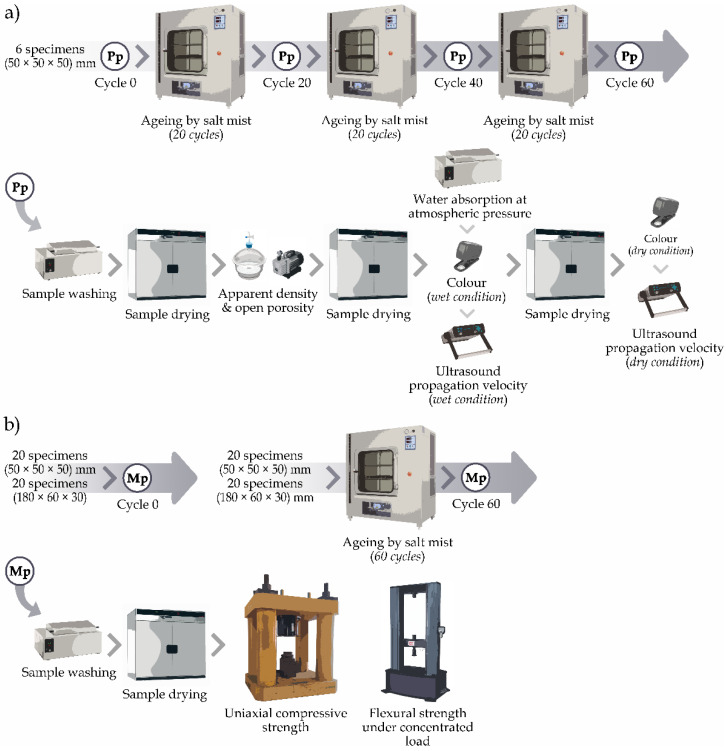
General scheme of the research methodology: (**a**) physical properties and (**b**) mechanical properties. Pp = Physical properties, Mp = Mechanical properties.

Physical properties were determined at the beginning, with the unaged specimens (cycle 0) and subsequently were obtained on the same set of initial specimens, every 20 cycles (at 20, 40 and 60 cycles) (Figure 3a). After each 20-cycle period, the samples were washed and soaked for 24 h before the physical properties were determined. The washing procedure described in the standard was not followed, as it was not intended to completely remove the accumulated salt, but to alter as little as possible the conditions to which the samples would have been subjected if the 60 cycles had been carried out in an uninterrupted manner. The mechanical properties were determined on one group of specimens without ageing (cycle 0) and on another after being subjected to 60 cycles of salt mist (Figure 3b). The specimens used to determine the mechanical properties after the 60 cycles of ageing were washed using the same procedure as described above.

The rate change was determined for the physical properties: apparent density (AD), open porosity (OP), water absorption at atmospheric pressure (WA) and ultrasound P-wave velocity propagation (PWV) and for the mechanical properties: uniaxial compressive strength (UCS) and flexural strength under concentrated load (FS). In all cases it was calculated according to Equation (1).

(1) $$\Delta P=\frac{P_{0}-P_{N}}{P_{0}}\times 100\;\left(\%\right)$$

where *P*_0_ corresponds to the physical property (AD_0_, OP_0_, WA_0_ or PWV_0_) or mechanical property (UCS_0_ or FS_0_) of the fresh rock and *P_N_* to the physical property (AD*_N_*, OP*_N_*, WA*_N_* or PWV*_N_*) or mechanical property (UCS*_N_* or FS*_N_*) obtained after *N* salt mist cycles.

The colour difference corresponds to the Euclidean distance between two points in CIEL*a*b* space, which was calculated by Equation (2):

(2) $$\Delta E_{Lab\;}=\sqrt{{\left(\Delta L^{*}\right)}^{2}+{\left(\Delta a^{*}\right)}^{2}+{\left(\Delta b^{*}\right)}^{2}}$$

where ΔL*, Δa* and Δb* are the difference between the parameters (L* = lightness, a* = red–green, b* = blue–yellow) measured on fresh rock and after *N* salt spray cycles and are calculated as follows: ΔL* = (L*_N_ − L*_0_), Δa* = (a*_N_ − a*_0_) and Δb* = (b*_N_ − b*_0_). Values of Δ*E_Lab_* > 2 indicate that it is possible to notice colour differences, while with values of Δ*E_Lab_* > 5, the colours are perceived as different [62].

## 4. Results and Discussion

### 4.1. Petrographic, Mineralogical and Chemical Analysis

#### 4.1.1. Petrographic Analysis

**Figure 4:**
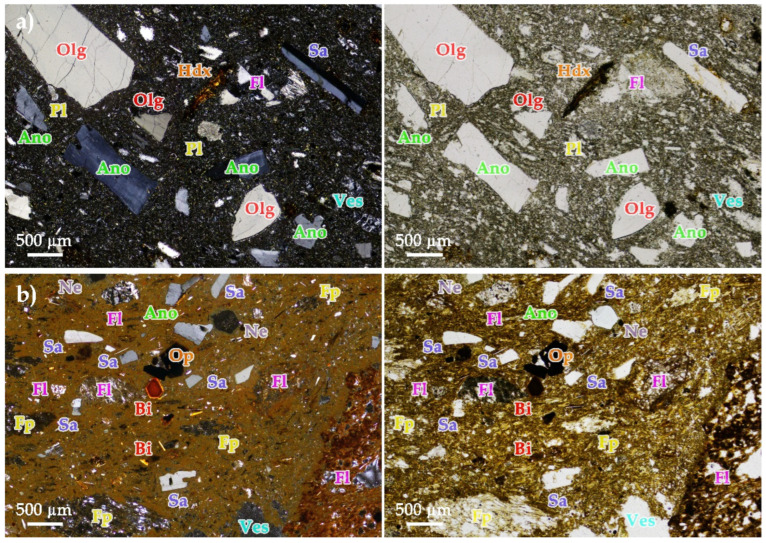
Microphotographs (crossed polarised—(**left**); plane polarised—(**right**)): (**a**) Lomo Tomás de León (LT) and (**b**) Marrón de Abades (MR). Ano = Anorthoclase, Bi = Biotite, Fl = Lytic fragment, Fp = Pumice fragment, Hdx = Hydroxides, Ne = Nepheline, Olg = Oligoclase, Pl = Plaglioclase altered, Sa = Sanidine, Ves = Vesicles.

LT ignimbrite is an extrusive igneous rock with an eutaxitic texture consisting of sub-angular to sub-rounded lithic fragments of trachytic/phonolitic composition and various minerals dispersed in a cinereous matrix with vitreous characteristics (Figure 4a). Oligoclase is the most abundant mineral, followed by alkali feldspars, sanidine and anorthoclase, all in the form of phenocrysts. Smaller amounts occur as microphenocrysts, augite and sporadically phlogopite and plagioclase of apparently more calcic composition with evidence of alteration and opacities, possibly hematite. LT ignimbrite has abundant iron hydroxides and its vesicles are perimetrically occupied by zeolites.

MA ignimbrite is an extrusive igneous rock of eutaxitic texture consisting of pumice fragments, lithic fragments of trachytic and phonolitic composition (most abundant) and volcanic glass fragments contained in a cinereous matrix (Figure 4b). The pumice fragments are mostly deformed and flattened, indicating a preferential flow orientation, while the rest of the lithics are angular. The most common phenocrysts are alkali feldspars (anorthoclase and sanidine). In lesser proportion, aegirine–augite, nepheline and biotite crystals, and rarely sphene, hornblende. Not very abundant, opaque (possibly hematite). Small, scattered and very abundant iron hydroxides are found in the matrix.

#### 4.1.2. X-ray Fluorescence (XRF)—Chemical Analysis

The results of the chemical analysis, expressed as percentage by weight of the major element oxides, are shown in Table 1. The weight percentages of most of the components, with the exception of aluminium oxide (Al_2_O_3_), titanium oxide (TiO_2_) and manganese oxide (MgO), vary by more than 5% from one ignimbrite to another, indicating a difference in the chemical composition of the ignimbrites studied. The most notable differences are in magnesium oxide (MgO), calcium oxide (CaO) and phosphorus oxide (P_2_O_5_), with the weight percentages of these components in the MR ignimbrite being more than double those measured in LT ignimbrite. The loss on ignition (LOI) is also much higher in the MR ignimbrite, indicating that this ignimbrite has a higher volatile content.

Projecting the content of alkaline minerals (Na_2_O and K_2_O) against the content of silicates (SiO_2_) in a TAS diagram [63], it can be deduced that LT ignimbrite has a trachytic composition, belonging to the alkaline series, while MR ignimbrite is in the zone of the diagram indicating a phonolitic composition, corresponding to the magmatic hyperalkaline series.

#### 4.1.3. X-ray Diffraction (XRD)—Mineralogical Analysis

The results of the mineralogical analysis are presented in Table 2. The most common mineral found in LT ignimbrite is oligoclase (Olg), followed by sanidine (Sa) and anorthoclase (Ano). Together, these three minerals account for more than 90% of the modal share of this ignimbrite. The other minerals that make up LT ignimbrite are augite (Aug), phlogopite (Phl), nepheline (Ne) and sodaline (Sdl), although the last three are rare. MR ignimbrite is mainly composed of anorthoclase (Ano) with a modal proportion of more than 70%. Sanidine (Sa), augite (Aug) and nepheline (Ne) are the other minerals found in this ignimbrite. LT ignimbrite has the same mineralogical composition as the MR ignimbrite, plus oligoclase (Olg), the presence of which is one of the most notable differences, along with the modal proportion of anorthoclase (Ano), which is much higher in MR ignimbrite than in LT ignimbrite. Nepheline (Ne), present in MR ignimbrite and rare in LT ignimbrite, is another distinguishing feature.

#### 4.1.4. Scanning Electron Microscopy

**Figure 5:**
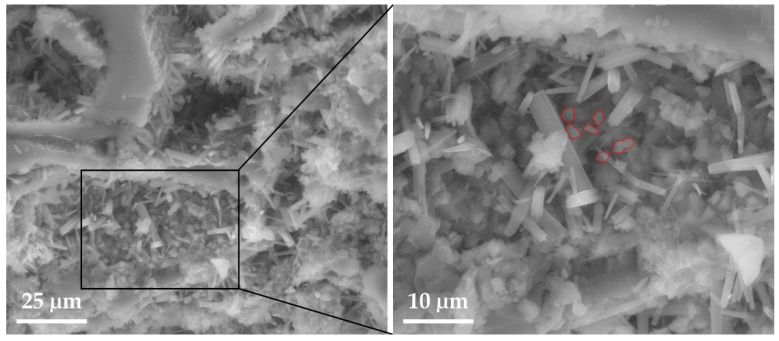
SEM image: Marrón de Abades (MR) with identification of NaCl crystals (red line).

The composition and texture of the ignimbrites studied make it difficult to see the salt crystals. In LT ignimbrite, the smaller crystals can be seen adhering to the larger minerals, and the cubic geometry typical of NaCl crystals are barely visible, possibly due to their partial breakage by the pressure exerted on the walls of the cavities. In MR ignimbrite (Figure 5), the salt has accumulated in a large pore and cubic salt crystals are now visible at the bottom of the cavity.

### 4.2. Physical Properties

#### 4.2.1. Apparent Density and Open Porosity

LT ignimbrite had an apparent density of 2.10 g/cm^3^ at the start and 2.09 g/cm^3^ after 60 salt mist cycles. This decrease, which represents a 0.5% change in apparent density, is negligible. In MR ignimbrite, the same apparent density was obtained at the beginning and end of the ageing test, and although the apparent density increased by 0.6% at cycle number 20, this was not maintained in subsequent cycles. This is also a non-significant variation. Similar results have been found in the literature where the variation in apparent density is less than 1%. [33,39].

The open porosity of the unaged LT ignimbrite was 15.3% and it increased to 19.0% after salt mist cycles. MR ignimbrite showed a similar behaviour as its open porosity also increased with respect to the initial open porosity, although the final variation of LT ignimbrite (24%) was practically double that of MR ignimbrite (13%). It should be noted that the open porosity of MR ignimbrite increased as it was exposed to the cycles, so that the greatest variation corresponded to the last cycle, whereas the open porosity decreased in cycle 60 with respect to cycle 40 in LT ignimbrite. The most notable variation occurred, close to 30%, in cycle 40. The average values of apparent density and open porosity obtained initially and after every 20 cycles of salt mist ageing are shown in Table 3.

The effect of salt mist ageing on open porosity varied according to the characteristics of each building stone, and granites can be found in which the open porosity after ageing is equal to the initial one, and others in which the open porosity increases by 50% [33,39,40], the latter being the most common behaviour in this type of material.

#### 4.2.2. Water Absorption

Water absorption at atmospheric pressure decreased in the two ignimbrites, with the final variation (cycle 60) being higher in LT ignimbrite (2.2%) than in MR ignimbrite (1.5%). In both cases, the maximum variation occurred in cycle 40, where water absorption decreased by 3.1% for LT ignimbrite and 2.6% for MR ignimbrite. The results show that there is a slight decrease in water absorption, but the variation obtained, either at the maximum (cycle 40) or at the end of ageing (cycle 60), is not very significant, as it hardly exceeds 3% (Table 3).

The increase in water absorption after salt spray aging has been reported previously by other authors [30], suggesting that this behaviour may be common in some masonry. However, in this study, as in others, water absorption at atmospheric pressure is either unchanged or increased, in some cases by more than 70% [33,39,40].

#### 4.2.3. Colourimetry

**Figure 6:**
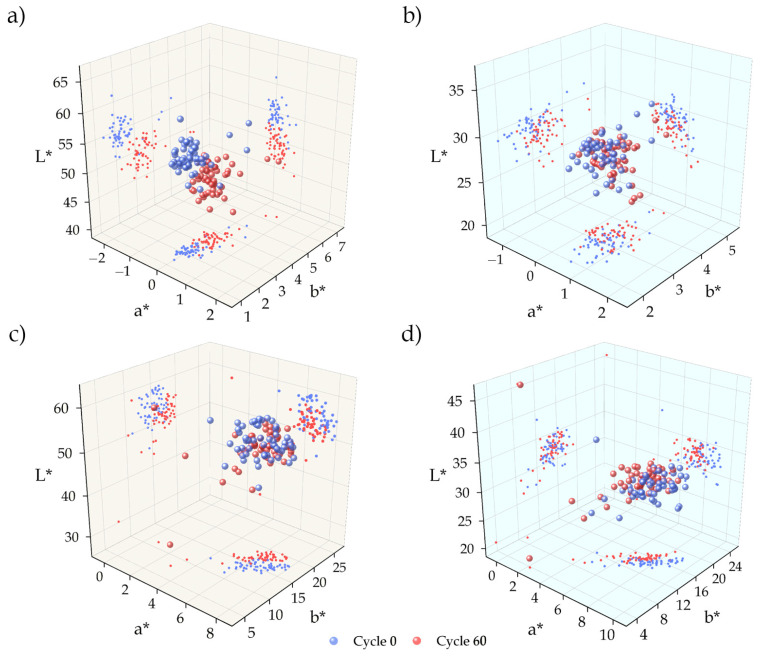
Scatter plots for colour parameters (L* = black–white, a* = red–green value and b* = blue–yellow value) before (original colour) and after 60 cycles of salt mist, in dry (**left**) and wet conditions (**right**): (**a**,**b**) Lomo Tomás de León (LT) and (**c**,**d**) Marrón de Abades (MR).

The colour difference of LT ignimbrite between its initial state and after ageing was Δ*E_Lab_* = 5.22 in dry conditions and Δ*E_Lab_* = 1.37 in wet conditions (Equation (2)). The difference in colour under dry conditions (Δ*E_Lab_* > 5) means that the colour of the specimens before and after ageing has been modified to such an extent that they can be perceived as different colours. The parameters that have changed the most were b* (blue/yellow) and L* (black/white). When the samples were subjected to 60 cycles of salt mist, the b* parameter decreased, which means that the colour tends to be bluer, while L* parameter decreased, which means that the colour is darker (Figure 6a). The behaviour of these parameters was similar in wet conditions, but their variation was much smaller (Figure 6b) and with a total colour difference (1 < ∆*E_Lab_* < 2) the colour difference can hardly be distinguished.

MR ignimbrite has a dry colour difference Δ*E_Lab_* = 3.48 and a wet colour difference Δ*E_Lab_* = 1.18 (Equation (2)). In dry conditions, and according to the colour difference obtained (2 < ∆*E_Lab_* < 3.5), it is possible to perceive the difference between its initial colour and its colour after ageing. In this ignimbrite, the most altered parameters were a* (red/green) and L* (black/white). Both parameters increased, which means that the colour measured after ageing tends to be redder (a*) and black (L*) than the initial colour (Figure 6c). The differences in parameter a* appears to be maintained, in wet conditions, but there is little variation in the remaining parameters (Figure 6d). The colour difference in wet conditions can hardly be perceived (1 < ∆*E_Lab_* < 2).

**Figure 7:**
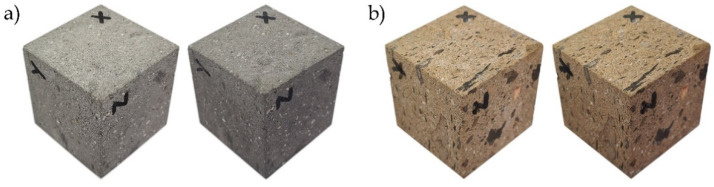
Variation of colour in dry condition before (**left**) and after (**right**) 60 cycles of salt mist: (**a**) Lomo Tomás de León (LT) and **(b**) Marrón de Abades (MR).

The difference in colour between the initial and aged state was greater and more noticeable in dry conditions for both LT and MR ignimbrites. The difference in the a* and b* parameters was not significant enough to be seen with the naked eye, but it was evident that the colour of both ignimbrites has darkened (L* decreases) (Figure 7). The colour change was more pronounced for LT ignimbrite; in fact, the initial and final colours (after 60 cycles) were practically perceived as two different colours (Figure 7a), as indicated by the ∆*E* value. For MR ignimbrite, the colour change was not obvious, but it was enough to see the difference between the initial colour and the colour after ageing (Figure 7b), in accordance with the ∆*E* value obtained.

The individual behaviour of the colorimetric parameters was different for each type of stone, so the effect of fog ageing on them was not always the same. However, it is clear that this ageing process modified the colour, with ∆E values varying from 4% to 12% depending on the study [33,44].

#### 4.2.4. Ultrasound Propagation Velocity (PWV)

**Figure 8:**
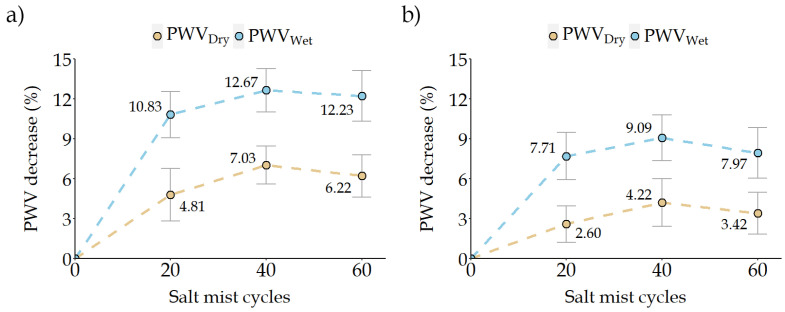
Variation of the ultrasound propagation velocity (PWV) after 60 cycles of salt mist, in dry and wet conditions: (**a**) Lomo Tomás de León (LT) and (**b**) Marrón de Abades (MR).

The ignimbrites studied showed a decrease in ultrasound velocity with salt mist ageing. LT ignimbrite had an initial ultrasound velocity of 3752.8 m/s in dry conditions and 4045.0 m/s in wet conditions, and after 60 cycles of salt mist, the values were 3519.3 m/s and 3550.1 m/s, respectively. MR ignimbrite had an initial ultrasound velocity of 3380.0 m/s in dry conditions, which increased to 3533.6 m/s after ageing while in wet conditions changed from an initial ultrasound velocity of 3533.6 m/s to 3252.1 m/s after ageing. Although the final variation of the LT ignimbrite (dry 6.22%/wet 12.2%) was higher than that of the MR ignimbrite (dry 3.4%/wet 8.0%), both ignimbrites showed similar behaviour, which was reproduced in both dry and wet conditions (Figure 8a,b). During the first 20 salt mist cycles, the velocity of the ultrasound decreased at a faster rate. Between cycle 20 and cycle 40, the ultrasound velocity continued decreasing, but at a slower rate. The maximum variation was obtained in cycle 40, which is more pronounced in wet conditions. From cycle 40 onwards, the ultrasound velocity tended to stabilise, and by cycle 60, it did not decrease but increased slightly, which may indicate that the deterioration occurs mainly in the first 40 cycles.

The variation obtained in the ultrasound velocity (under dry conditions) was similar to that obtained by other authors, with a decrease ranging from 3% to 9% [33,35]. There are also cases where the ultrasound velocity increases after ageing cycles; however, this behaviour is rare [40].

### 4.3. Mechanical Properties

#### 4.3.1. Uniaxial Compressive Strength

**Figure 9:**
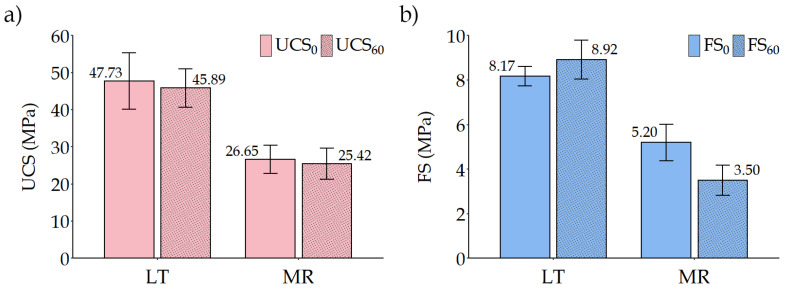
Mean value comparison before and after 60 salt mist cycles: (**a**) uniaxial compression strength (UCS) and (**b**) flexural strength (FS).

The initial compressive strength was 47.73 MPa for LT ignimbrite and 26.65 MPa for MR ignimbrite. After 60 cycles of salt mist ageing, the compressive strength of both ignimbrites decreased slightly: 45.89 MPa for LT ignimbrite and 25.42 MPa for MR ignimbrite. The variation obtained for LT ignimbrite and MR ignimbrite using Equation (1) was 3.8% and 4.6%, respectively. Figure 9a shows the average compressive strength, including the standard deviation of the results for each ignimbrite, without and with ageing.

The results obtained show a slight decrease in compressive strength, with an average variation of 4.2%. According to the available literature, these ignimbrites showed a very small variation compared to other types of building stones, where limestones and sandstones can suffer a decrease in compressive strength of 31% after salt mist ageing [35], granites between 7% and 62% [39] and trachytes a decrease close to 25% [40].

#### 4.3.2. Flexural Strength

The flexural strength showed a very different behaviour in each of the two ignimbrites. For the LT ignimbrite, the flexural strength was 8.17 MPa for the unaged stone and 8.92 MPa for the ageing stone, whereas for MR ignimbrite, the initial flexural strength was 5.20 MPa and the final flexural strength was 3.50 MPa after the 60 salt mist cycles. In terms of variation, calculated using Equation (1), this means that the LT ignimbrite experienced an increase of 9.2%, while the MR ignimbrite experienced a decrease of almost 33%. The average flexural strength values obtained for each ignimbrite, together with their standard deviation, are shown in Figure 9b.

The variation in flexural strength after salt spray ageing was similar to that found in other studies, where the flexural strength usually decreases by around 10% to 30% [29,30]. The increase in flexural strength that occurred in LT ignimbrite was an unusual behaviour, but it also occurred in these studies, and although the variation was close to 9%, it cannot be concluded that it is a significant increase due to the scatter of the results.

## 5. Conclusions

The apparent density of both ignimbrites remains constant throughout the ageing process, mainly since no material loss has been detected. The dry mass of the samples does not vary (no loss of mass) and, although the saturated mass and the submerged mass increase with the number of cycles, they do so in similar proportions, presenting the same apparent volume and, consequently, practically the same apparent densities in all the cycles in which they have been determined.

Open porosity is by far the property with more change, increasing significantly after 60 salt mist cycles. LT ignimbrite has the lowest open porosity and has the highest variation, while MR ignimbrite, being more porous, has lower variation. The difference in behaviour between these two ignimbrites is due to the fact that the salt crystals in LT ignimbrite had less space to grow, due to the smaller pore size of this sample, resulting in a higher crystallisation pressure and consequently more damage. In MR ignimbrite, some crystals did not grow large enough to occupy the entire space of the larger cavities, so these crystals did not cause any damage because they did not exert any pressure. This is evidence that, in addition to porosity, pore size plays a fundamental role in ignimbrite durability.

Primarily, open porosity increases because the crystallisation of salts causes certain pores to increase in size. Fractures and micro-fractures are also affected by this phenomenon and, in addition to increasing in length or spacing, new fractures may be created. To determine open porosity, as required by the relevant standard, samples are subjected to a vacuum, which removes air from the voids and allows water to enter the porous structure of the stone. However, water absorption takes place at atmospheric pressure and water cannot always occupy the voids created by ageing, while other voids, new or existing before ageing, are inaccessible because salt crystals obstruct or prevent water access. This may explain why water absorption decreases while open porosity increases.

The ultrasound propagation velocity decreases, indicating that salt spray ageing has altered the porous structure of the studied ignimbrites. As there are more empty spaces, the wave encounters a greater volume of air as it travels, slowing its velocity.

With the exception of apparent density, all other physical properties are affected after 60 salt mist cycles, and although the degree of change varies from property to property, they all show similar behaviour. Apart from the fact that open porosity increases and the other properties decrease, the greatest change occurs in the first 40 cycles, after which the effect seems to diminish and recover slightly, with a slight tendency to stabilise. This reduction in the variation of physical properties from cycle 40 onwards may be due to the accumulation of salt crystals in the voids. This would explain why the open porosity decreases and the water absorption and ultrasound velocity increase compared to the properties determined at cycle 40.

The colour analysis carried out shows that there is a colour change in the ignimbrites after being subjected to 60 cycles of salt mist. However, although this ageing process may have altered the colour of the samples, we believe that the colour change is more likely to be related to the different drying stages that the samples were subjected to before the start of each test to determine their physical properties.

In general, taking into account the scatter of the results obtained in the compression and flexural tests, it cannot be concluded that salt spray ageing causes a significant variation in the mechanical properties of LT ignimbrite. The same is true for MR ignimbrite, at least as far as its compressive strength is concerned, since in this case, the variation in flexural strength is significant, showing a decrease of more than 30% after 60 salt mist cycles.

Salt mist ageing test is probably one of the tests that has undergone the most adaptations and modifications during its performance. Cycle number, drying and spraying times, salts concentration, sample size, washing time, etc., make it difficult to make an objective comparison between the various studies available in the literature.

Only two ignimbrites have been studied. A larger collection with a wide range of porosity values will provide a better understanding of the salt spray effect. The small number of cycles is also always problematic when trying to understand how the stones will behave over decades.

This research is the first study of the effect of salt spray ageing on physical and mechanical properties of these two ignimbrites and, to our knowledge, is the first study in this lithology. The results are the first approach to assess the behaviour of these materials in coastal environments and provide some clues for the necessity of using protective products.

## Figures and Tables

**Table 1:** Major element oxides of ignimbrite samples (oxides are expressed as wt %).

Code	SiO_2_	Al_2_O_3_	Fe_2_O_3_	MnO	MgO	CaO	Na_2_O	K_2_O	TiO_2_	P_2_O_5_	LOI
LT *	62.99	17.07	4.19	0.21	0.46	0.45	6.36	5.38	0.90	0.05	1.94
MR	58.46	17.75	3.62	0.20	1.25	1.23	7.63	4.63	0.88	0.12	5.03

LT = Lomo Tomás de León; MR = Marrón de Abades. * Results from Valido et al. [38].

**Table 2:** Semi-quantitative mineralogical composition results obtained via X-ray diffraction (XRD).

Code	Ano	Aug	Ne	Olg	Phl	Sa	Sdl
LT	•••	••	•	••••	•	•••	•
MR	••••	••	••	◦	◦	•••	◦

LT = Lomo Tomás de León; MR = Marrón de Abades. Minerals: Ano = Anorthoclase, Aug = Augite, Ne = Nepheline, Olg = Oligoclase, Phl = Phlogopite, Sa = Sanidine, Sdl = Sodalite [64]. Symbol: ◦ = Not present, • = Scarce (≤2%), •• = Present (2–10%), ••• = Abundant (10–40%), •••• = Very abundant (≥40%).

**Table 3:** Effect of salt mist ageing on the apparent density, open porosity and water absorption.

Code	No. of Salt Mist Cycles	AD (g/cm^3^)		OP (%)		WA (%)	
		Mean	Std. Dev	Mean	Std. Dev	Mean	Std. Dev
LT	0	2.10	0.03	15.32	1.05	7.15	0.80
	20	2.10	0.03	17.55	1.64	7.06	0.81
	40	2.09	0.03	19.69	1.01	6.93	0.79
	60	2.09	0.03	19.00	0.93	6.99	0.78
MR	0	1.68	0.03	30.04	1.07	16.45	1.30
	20	1.69	0.03	32.41	1.63	16.36	1.31
	40	1.68	0.03	33.35	1.27	16.03	1.33
	60	1.68	0.03	33.94	1.32	16.20	1.33

LT = Lomo Tomás de León, MR = Marrón de Abades, AD = Apparent density, OP = Open porosity and WA = Water absorption.

## Data Availability

The data presented in this study are available on request from the corresponding author.
